# The Current State and Future Directions of Swallowing Care in Amyotrophic Lateral Sclerosis

**DOI:** 10.1007/s40141-023-00396-5

**Published:** 2023-04-18

**Authors:** Tabitha H. Kao, Bridget J. Perry

**Affiliations:** 1 MGH Institute of Health Professions, Boston, MA, USA

**Keywords:** Difficulty swallowing, Dysphagia, ALS

## Abstract

**Purpose of Review:**

Difficulty swallowing (dysphagia) is of great concern to patients with ALS as its complications can increase mortality and reduce the quality of life. This review aims to provide an overview of the recent developments and the current state of assessment, treatment, and management of dysphagia in ALS.

**Recent Findings:**

The optimal timing of assessment, treatment, and management of dysphagia may be early in the ALS disease process, even before the dysphagia occurs. There is wide heterogeneity in SLP practice patterns for the management of dysphagia.

**Summary:**

Dysphagia is common and debilitating; however, for various reasons, there is no clear consensus on how best to manage dysphagia in this population. Future work centered around predicting swallowing decline and improving interventions aimed at prolonging swallowing function in the early stages of the disease process may promote improved dysphagia care.

## Introduction

Over the past 5 years, research has markedly advanced our understanding of the assessment and treatment of devastating swallowing impairments that impact most individuals living with amyotrophic lateral sclerosis (ALS) [1]. While the onset of dysphagia typically occurs sooner for those whose symptoms begin in the bulbar regions, the timing of dysphagia development and progression varies widely among individuals. Dysphagia is of great concern to those affected by ALS as its complications, such as aspiration pneumonia, have been found to increase mortality [2] and reduce the quality of life [3].

This review aims to provide an overview of ALS-related dysphagia consisting of the current state of healthcare practice, the timing of assessments and interventions, the development and validation of examinations, the effectiveness of exercises, and the benefits of oral and non-oral nutritional management methods. It begins with a brief background of ALS-related dysphagia, followed by discussions of (1) commonly used and new assessment methodologies, (2) rehabilitative and compensatory interventions, and (3) recommendations for future research. Table 1 summarizes the studies dated from 2017 to 2022 used in this review, categorized by the levels of evidence classification system described by Dang et al. [36].

## Swallowing Function in ALS

Studies estimate that up to 92% of individuals with ALS experience dysphagia [1, 17, 37]. ALS-related dysphagia can result in physical complications, including aspiration pneumonia [2]; malnutrition and dehydration are predictors of survival in this population [38, 39]. Dysphagia can negatively impact the quality of life, including sleep quality, mental health, social interactions, and eating desire [40]. Additionally, it can increase general fatigue, eating duration, fear of eating-related complications, eating-related burden, and difficulty with food selection [3].

Much of the literature has focused on the oropharyngeal physiology of dysphagia. Signs and symptoms may differ depending on the onset type of ALS; individuals with bulbar onset often have earlier and more difficulty swallowing than those with spinal onset. Oral phase impairments include reductions in tongue speed, coordination, and range of motion; reduced tongue base retraction; reduced bolus preparation; the presence of oral bolus holding behavior; increased oral transit time; impaired anterior-posterior bolus transport; increased oral residue; and delayed initiation of the pharyngeal phase [2, 18, 19, 20, 41]. In the pharyngeal phase, common impairments include reduced anterior hyoid excursion; reduced laryngeal elevation; reduced pharyngeal constriction; increased vallecular and pyriform sinus residue amount; and increased number of aspiration and penetration events on liquids [2, 18, 41]. However, upper esophageal sphincter opening generally appears intact, even in the later stages of ALS [2].

Respiratory and swallowing functions in ALS are closely related, and weakness in the respiratory muscles can impair aspiration-protective mechanisms like coughing [31, 42]. Studies have found that among individuals with ALS, participants considered unsafe swallowers had reduced voluntary and reflexive cough effectiveness compared to safe swallowers, placing them at high risk for aspiration-related complications, including pneumonia [26, 42].

## Assessment

Assessing swallowing through screens or evaluations is the first step toward developing a patient-specific care plan. Screens are quicker and easier to administer but often need more detailed information to make a treatment plan. Because of their simplicity, their primary purpose is to indicate if there is a need for further evaluation [15, 30, 43]. Evaluations are typically more time and resource extensive than screens. An evaluation aims to determine the presence and severity of dysphagia so that clinicians can develop comprehensive management or treatment plans. Evaluations may use easily accessible clinical materials or specialized instrumental assessments like videofluoroscopy (VFSS) or fiberoptic endoscopy (FEES). Instrumental assessments can determine (1) physiologic impairments present during swallowing, (2) consistencies safest for oral intake, and (3) compensatory strategies that best minimize aspiration and maximize swallow efficiency [15, 43]. Additionally, healthcare teams can use them to educate individuals and their caregivers about their swallowing impairment.

Current literature suggests that the indications and methods for and timing of swallowing assessments vary widely among providers and healthcare systems, resulting in inconsistent care within and between patients. Below, we summarize widely used swallowing screens and evaluations for individuals with ALS. Table 2 summarizes the psychometric properties of the tools included in this review. Note that some assessment tools need validation in the ALS population; thus, their sensitivity and specificity will not be available. In addition to swallowing, individuals with ALS have other bulbar impairments of salivation and speech. Yunusova et al. [30] provide a comprehensive overview of bulbar-related assessments in individuals with ALS.

## Screens

The 3-oz water test and the Eating Assessment Tool-10 (EAT-10) are two commonly used screening tools that recently underwent validation studies in individuals with ALS. Both screens are quick and easy to administer, but the 3-oz water test is performance-based, whereas the EAT-10 is patient-reported. While task performance-based measures may be more objective, patient-reported outcomes may be more clinically meaningful.

Two studies have compared the 3-oz water test’s ability to detect aspiration or penetration to the penetration-aspiration scale (PAS) during VFSS. In a study with 31 participants with motor neuron disease including ALS (Focht), Garand et al. [15] found the screen detected aspiration with a sensitivity of 80% and a specificity of 33%. However, in their study with 212 participants with ALS, Donohue et al. [44] found that the 3-oz water test detected aspiration with a sensitivity of only 55% and a specificity of 72%. Based on their respective findings (Focht), Garand et al. recommend evaluation via VFSS if the individual does not pass the 3-oz water test, whereas Donohue et al. recommend against using it to screen for aspiration.

The EAT-10 is a self-reported questionnaire that probes the patient’s perspectives about their degree of swallowing impairment; the higher the score, the more severe the patient perceives their swallowing impairments to be [46]. In a group of 70 participants with ALS, Plowman et al. [43] compared the EAT-10 to the PAS during VFSS. They found that an EAT-10 score of 3 detects penetration and aspiration with a sensitivity of 88% and a specificity of 57%. Additionally, a score of 8 detects aspiration with a sensitivity of 86% and specificity of 72%. A later study by Donohue et al. [13] compared EAT-10 to the Dynamic Imaging Grade of Swallowing Toxicity scale in 273 participants with ALS. They found that an EAT-10 score of 3 detects mild dysphagia with a sensitivity of 77% and a specificity of 53%. Additionally, a score of 7 detects moderate dysphagia with a sensitivity of 81% and a specificity of 66%.

In addition to the 3-oz water test and EAT-10, Perry et al. [21] recently developed a clinical prediction model specific to ALS using commonly collected clinical variables to estimate a patient’s 3-month, 6-month, and 12-month risk of developing dysphagia. This model allows the healthcare team to input variables such as time since symptom onset and ALS functional rating scale total and subscale scores into an online calculator to generate a dysphagia-risk score. When using this model, the authors suggest that further swallowing evaluations may provide added value when the 3-month risk scores range from 15 to 50%.

## Evaluations

In clinical practice, SLPs use a variety of evaluations to assess swallowing function in individuals with ALS [14•]. In a survey by Epps et al. [14•] from 2016 to 2017, tests used to diagnose dysphagia included clinical swallowing assessments, instrumental assessments, swallowing screens, and patient surveys. Clinical swallowing assessments, which likely included oral mechanism examinations and bolus trials, were the most commonly administered assessment among 49 SLPs throughout the USA. Additionally, Plowman et al.’s 2017 survey [22] listed routinely collected clinical outcomes such as weight and forced vital capacity. In both of these studies, the authors observed that the swallowing tests and outcomes collected varied among clinicians. Here, we have focused on assessments commonly used swallowing assessments in individuals with ALS.

In a recent survey, the ALSFRS-R was the most consistently administered metric for tracking ALS disease progression [22]. The ALS-FRS is a 12-item patient-report scale about bulbar, fine motor, gross motor, and respiratory functions [47]. The bulbar subscale includes one question directly targeting swallowing function; lower scores indicate more significant impairment. Recently, a study by Chapin et al. [12], including 201 participants with ALS, found that an ALSFRS-R swallowing item score less than or equal to 3 was associated with penetration or aspiration with a sensitivity of 79% and a specificity of 60%.

The Center for Neurological Study Bulbar Function Scale (CNS-BFS) is a more recent patient-report scale rating the speech, swallowing, and salivation of individuals with ALS [25]. Higher scores are indicative of perceived worse impairment. The CNSBFS is well-correlated with the ALSFRS-R bulbar subscale, with a correlation coefficient of 0.90. Findings suggest that a score of 43 and above may indicate bulbar impairment. There are currently no validation studies comparing CNS-BFS to an instrumental swallowing assessment [30].

Unlike clinical assessments, instrumental assessments like VFSS and FEES allow SLPs to directly visualize the oropharyngeal structures involved in swallowing. As such, they may give clinicians more confidence in identifying swallowing impairments, selecting and probing appropriate compensatory strategies such as diet modifications, and determining the need for feeding tube placement [15, 30]. Additionally, they can serve as visual aids when educating patients and caregivers about aspiration risks. VFSS allows clinicians to observe physiological movements of the oropharyngeal structures from the front and lateral views using fluoroscopy. In contrast, FEES provides a superior view of the larynx and pharynx, including vocal fold appearance and movement, tissue integrity, and secretion management.

However, despite their benefits, research suggests that instrumental evaluations are not being utilized consistently for assessment in this population [15]. Reasons that healthcare professionals may not refer individuals with ALS for instrumental evaluation include a perceived lack of clinical utility due to the progressive nature of ALS and related dysphagia or new information not already obtained from clinical assessments [22]. Accessibility may be a barrier to VFSS as the facility may not be able to administer it; additionally, the patient may need an additional appointment to undergo the test. As for procedures, cleaning protocols required after FEES evaluations and scheduling limitations with VFSS may confine the number of assessments administered. Regardless of these barriers and the lack of formal guidelines regarding the timing of instrumental assessment, the literature suggests that SLPs should conduct instrumental evaluations early and repeatedly based on dysphagia indications [18, 27], with one study recommending VFSS at 6-and 12-month post-bulbar symptom onset. Frequent assessments allow SLPs and patients to comprehensively understand the progression of swallowing dysfunction.

To describe instrumental evaluation findings, clinicians may utilize standardized metrics. One specific to VFSS is the Modified Barium Swallow Impairment Profile (MBSImP), comprised of seventeen components categorized by the oral, pharyngeal, and esophageal phases [48]. Unfortunately, there appear to be no published studies that use MBSImP as recommended to characterize the physiologic impairments of swallowing function in ALS. However, Murono et al. [41] describe oral and pharyngeal phase impairments in 19 participants following an MBSImP-like framework. While they did not follow the administration protocol nor examine esophageal phase deficits, they found that those with bulbar symptoms of ALS had impaired bolus preparation/mastication and the initiation of the pharyngeal swallow during the oral phase. Additionally, participants with and without bulbar symptoms had impairments in bolus transport/lingual motion and oral residue. For the pharyngeal phase, those with bulbar symptoms had impairments in laryngeal elevation, anterior hyoid excursion, and tongue base retraction. Participants with and without bulbar symptoms had pharyngeal residue.

Murono et al. [41] also used the PAS to assess the incidence of penetration and aspiration observed on either VFSS or FEES in patients with ALS. The PAS is a clinician-rated scale that describes the location of penetration or aspiration, patient response, and airway clearance [49]. In their study, Murono et al. [41] found that among the 19 participants, those with bulbar symptoms had an average score of 1.50, and those without bulbar symptoms had an average score of 1.20. These participants were evaluated shortly after their ALS diagnosis, which may explain this low incidence of penetration and aspiration. Hence, the authors suggest that penetration and aspiration are uncommon in participants with and without bulbar symptoms at the initial diagnosis and that the onset of dysphagia occurs later in the disease process.

The Dynamic Imaging Grade of Swallowing Toxicity (DIGEST) is another tool used to standardize instrumental assessment findings. It is a clinician-rated scale rating safety and efficiency that, when combined, result in a total score that indicates the severity of pharyngeal phase impairment ranging from 0 to 4. A higher total score indicates higher dysphagia severity [50]. Plowman et al. [6] used the DIGEST to describe dysphagia severity in 48 individuals with early-stage ALS undergoing active and sham expiratory muscle strength training trials. Their study defined total scores greater than 1, safety scores greater than or equal to 3, and efficiency scores greater than or equal to 1 as indicative of swallowing impairment.

While not a direct instrumental assessment of swallowing function, tongue movements during swallowing [19, 20] measured by electromagnetic articulography and tongue strength [10, 23] measured with the Iowa Oral Performance Instrument (IOPI) have been explored as potential biomarkers for swallowing impairment in this population. These studies suggest that changes in tongue movements during swallowing are present prior to the onset of swallowing impairment [19] and that tongue movements during swallowing had strong correlations with oral stage swallowing impairments as measured using MBSImP, PAS scale, and with patient-reported swallowing function [20]. Additionally, reduced tongue strength was found to be associated with inefficient and unsafe swallowing [10, 23].

## Interventions

In their 2020 paper, Rogus-Pullia and Plowman [29••] call for a proactive approach to assessing and treating swallowing impairment in neurodegenerative disorders, including ALS, stating that current clinical practices are primarily reactive. Specifically, this approach requires the active collaboration of an interdisciplinary team, including SLPs at the time of ALS diagnosis, to help facilitate patient-specific care plans [29••]. In this section, we describe recent developments in interdisciplinary approaches to addressing dysphagia, some of which have promising implications as we move our practice forward.

## Medical Interventions

Few pharmaceutical options exist for addressing ALS-related dysphagia [31]; one potential medication is dextromethorphan/quinidine (Nuedexta). Research by Smith et al. [7] found that compared to the placebo group, participants who received Nuedexta showed improvements in all subscales of the CNS-BFS, and half had at least a 1-point improvement on the ALSFRS-R bulbar subscale. However, the authors did not find significant differences in clinician ratings of speech and swallowing function between those who took Nuedexta and those who did not. This result suggests that Nuedexta may result in noticeable but minor patient-perceived improvements in speech, swallowing, and salivation; however, it is unclear if it can effectively improve swallowing.

Limited research has explored using laryngotracheal separation surgery, which reroutes the trachea to an anterior neck stoma, to prevent aspiration in the ALS population. Surgery may relieve discomfort and distress associated with aspiration for some individuals. Additionally, surgery may allow individuals to maintain oral intake for an average of 1 to 2 years and reduce the need for sputum suctioning [34]. However, although surgery prevents aspiration, it does not address the underlying mechanisms of swallowing impairment; thus, deficits in swallowing efficiency may persist [31, 34]. Additionally, due to the redirection of air from the vocal folds, individuals lose the ability to voice and communicate verbally through the glottis. Thus, as a result of surgery, patients who undergo this procedure become dependent on alternative and augmentative forms of communication.

## Electrical Stimulation

In a recent survey [14•], 94% of SLPs reported that they would not use electrical stimulation (ES) for the ALS population. Indeed, ES may not be effective in neurodegenerative diseases like ALS due to underlying pathology [5]. For example, one study [5] examined the efficacy of pharyngeal ES in 20 participants with ALS and severe dysphagia. The participants were either part of the control group (standard speech therapy (ST)) or the intervention group (standard ST with pharyngeal ES). The control group focused on sensorimotor perception, postural changes, swallowing maneuvers, and modification of oral intake. The intervention group received treatment for 10 min across three consecutive days. While both groups improved in swallowing safety up to 3 weeks after the intervention, standard ST with pharyngeal ES was comparable to ST alone. In other words, pharyngeal ES did not add any additional benefits.

## Exercise-based Interventions

Exercises focusing on muscle training and resistance for force generation [29••] have typically not been recommended for individuals with ALS since intensive exercise interventions theoretically might exhaust already weak muscles [31, 14•, 27]. For example, in Epps et al.’s survey [51], 85% of SLPs reported not using oromotor or laryngeal strengthening exercises as treatment in individuals with ALS. Additionally, a 2015 literature review by Plowman [51] found that instead of exercise, clinicians managing the ALS population focused primarily on safe swallowing strategies, diet modifications, feeding tubes, and compensatory strategies through adjusted postures. However, the 14 studies she analyzed indicated that early application of mildly-to-moderately intense limb and respiratory exercise might maintain function in individuals with ALS. Thus, Plowman suggests that mildly-to-moderately intense tasks may benefit swallowing.

Since respiratory and swallowing systems are closely related [26, 42], researchers have recently hypothesized that exercises aimed at respiratory musculature may simultaneously improve swallowing function [6, 51]. One such example is Expiratory Muscle Strength Training (EMST), an exercise routine centered around improving respiratory musculature strength. Participants in a single-case study [52] and a randomized controlled trial [6] demonstrated good tolerance of an 8-week home-based EMST program. In the randomized controlled trial, the authors found that the EMST group had maintained baseline global swallowing functioning and efficiency based on DIGEST scores compared to the sham group. The difference between the EMST and sham groups was significant for swallowing safety. Additionally, measures related to cough strength, like maximum expiratory pressure, improved in the EMST group. These studies suggest that EMST is safe and tolerable for individuals with early-to-middle stages of ALS and may be effective in improving swallowing function. However, it is unknown if EMST would be effective for those in the later stages or have severe dysphagia symptoms.

## Artificial Nutrition Interventions

Due to weight loss and malnutrition in ALS [38, 39], maintaining adequate nutrition is vital. Therefore, when swallowing becomes impaired, individuals with ALS may choose to maintain nutrition orally, artificially through the placement of a feeding tube, or through a combination of both. Before resorting to more invasive means of nutrition, the patient and healthcare team may opt for an oral route [9] with SLP support and individualized counseling through standard care or with a dietitian [8]. However, often these strategies are only effective temporarily. Eventually, patients and their healthcare teams may need to consider feeding tubes as ALS progresses, especially when there is severe dysphagia and significant weight loss [11].

Regardless of the method, patient preference for nutritional management should take precedence [32] as we currently lack well-established guidelines for best practices in the nutritional management of this population. When making nutrition recommendations, the healthcare team should consider patient-related factors including psychological adjustment, need for control, understanding of ALS and related complications, and psychosocial aspects of eating [28]. Although artificial nutritional management discussions should begin early in the disease process to reduce weight loss [9], research suggests that individuals have varying positive and negative responses to early discussions on this topic [28]. To encourage patient engagement in the nutritional management plan, healthcare teams should empower the individual through education about ALS and related swallowing impairments. Zarotti et al. [28] recommend teaching strategies that reduce stress, misconceptions, and fears about eating. To illustrate these strategies, Seeber et al. [24] describe their experience counseling 28 participants, which included tailoring caregiver education topics based on observations or participant concerns like feeding tube use and management discussions.

A survey by Plowman et al. [22] suggests that rates of feeding tube use vary widely between healthcare providers and systems, with greater than 70% of patients receiving feeding tubes in some ALS multidisciplinary clinics and < 15% in others. This wide variation partly results from a lack of clarity on the benefits and risks of nutritional management strategies on outcomes and quality of life [4]. Much of the research has focused on the potential benefits of feeding tubes, including weight maintenance and survival. Some studies suggest that feeding tubes provide a survival benefit [4], while others suggest feeding tubes reduce life expectancy [16]. Similarly, evidence supporting weight maintenance for those with feeding tubes is weak, with only a few controlled studies showing a nutritional advantage of feeding tubes [4, 38, 53]. Research in this area is complex due to the heterogeneity of the disease process and the challenges of overcoming selection bias associated with dysphagia management decisions [29••].

Although the optimal timing for placement is unclear, the literature indicates that surgery should occur before the individual is in the advanced stages of ALS or has excessively reduced forced vital capacity [11]. The 2012 guidelines from the European Federation of the Neurological Sciences suggest that individuals with worsening respiratory status should consider percutaneous endoscopic gastrostomy tubes (PEG) even in the absence of dysphagia [54], as once dysphagia develops, it may be too late for placement. Benefits of early placement include potentially maximizing nutritional status and allowing for active patient participation in nutritional management interventions [33].

## Conclusion

Shortcomings in care, uncertainty, and timing as well as practice variability affect the clinical assessment and management of dysphagia in individuals with ALS. Literature suggests that dysphagia assessment may be appropriate before patients even present with bulbar symptoms; however, most clinicians typically administer evaluations when symptoms manifest [29••]. Additionally, dysphagia evaluations vary widely among clinicians, and instrumental assessments are not used consistently, despite the high prevalence of swallowing dysfunction in this population. Although research using standardized instrumental evaluations of swallowing function is becoming increasingly more common in this population, it remains somewhat limited. As a result, our understanding of discrete changes in swallowing pathophysiology over the course of disease progression is limited [35]; thus, we cannot predict the progression of dysphagia. It is difficult to effectively and efficiently evaluate the impact of interventions aimed at slowing the onset or progression of dysphagia.

Likewise, many interventions directed at managing swallowing function in individuals with ALS remain primarily reactive rather than proactive [29•]. However, recent studies exploring the impact of EMST on swallowing function in individuals with early and middle stages of ALS [6] suggest that in the early stages of the disease process, (1) EMST may positively impact swallowing function, and (2) proactive, targeted exercise approaches may be promising.

In the absence of clinical best practices for dysphagia management in ALS, patient-centered care surrounding nutritional management strategies, including feeding tube placement and diet texture modifications, remains challenging, and practice patterns are highly variable. Further research in several areas of dysphagia assessment and intervention would advance our understanding and improve our management of swallowing impairment in ALS. To start, longitudinal studies using standardized instrumental assessment and measures would allow us to quantify swallowing changes over time as well as to describe and predict both meaningful and important changes in swallowing function. Determining meaningful and important change in swallowing function would also allow for us to more effectively and efficiently evaluate interventions aimed at prolonging swallowing function and begin to develop best practice guidelines for dysphagia management in this population. Additionally, further research centered around the development of proactive physiologically based exercise interventions to prolong swallowing function in the early stages of the disease process is greatly needed. Finally, in the absence of best practice clinical guidelines, research aimed at actively engaging patients in the dysphagia management decision-making process is critical to improving patient-centered care.

## Figures and Tables

**Table 1:** Summary of ALS-related studies published from 2017 to 2022 organized by levels of evidence

Summary of Current Studies (2017 – 2022)			
Study	Level of Evidence	Summary	Key Findings
**Level 1**			
Bond et al. (2019) [4]: retrospective cohort study with meta-analysis.	**lb:** Results reasonably consistent with literature; sufficient sample size for the study design; recommendations reasonable based on literature review.	Contains a retrospective analysis of an ALS cohort who had or did not have PEG tubes and a meta-analysis examining factors effecting length of survival.	PEG placement and a forced vital capacity ≥ 50% at the time of placement predict and increase survival duration because it stabilizes weight and body mass index.
Herrmann et al. (2022) [5]: prospective, randomized, parallel-group, controlled trial	**lb:** Results reasonably consistent with literature; fairly definitive conclusions; reasonable recommendations.	Explores the efficacy of the pharyngeal electrical stimulation compared to standard speech therapy on swallowing function in ALS participants with severe dysphagia.	The addition of pharyngeal electrical stimulation to standard speech therapy was comparable to standard therapy alone, meaning electrical stimulation did not have any benefits.
Plowman et al. (2019) [6]: single-center, double-blind, randomized controlled trial	**lb:** Results consistent with literature; sufficient sample size; made fairly definitive conclusions.	Discusses the results of the a mild-to-moderate intensity, 8-week, at-home expiratory muscle strength training (EMST) program in individuals with early-to-middle stage ALS.	EMST was safe, feasible, and tolerated in participants with early-to-middle stage ALS, resulting in higher maximum expiratory pressure, improved swallowing function.
Smith et al. (2017) [7]: randomized, blinded, crossover clinical trial	**lb:** Results reasonably consistent with literature; sufficient sample size; fairly definitive conclusions; made reasonably recommendations.	Examines the effect of Nuedexta on speech, swallowing, and salivation in individuals with ALS.	Nuedexta has significant but small improvements in speech, swallowing, and salivation according to patient-reported questionnaires, but not during clinical tasks.
Wills et al. (2019) [8]: unblinded, single-center pragmatic randomized controlled trial	**lb:** Sufficient sample size for the study design; fairly definitive conclusions.	Compares weight gain in individuals with ALS who either underwent received nutritional counseling under standard care or with a dietitian with or without an electronic health application.	Receiving nutritional counselling from a dietitian, with or without an electronic health application to aid with tracking weight, was comparable to standard care.
**Level 2**			
Essat et al. (2020) [9]: systematic review	**2b:** Results reasonably consistent with results; fairly definitive conclusions.	Discusses oral nutritional interventions, as opposed to enteral feeding, in individuals with ALS.	Although individuals with ALS appear to benefit from oral nutritional interventions, many of these effects are either not significant or long-term.
**Level 3**			
Borges et al. (2022) [10]: cross-sectional single-center observation study	**3b:** Results reasonably consistent with literature; some control; recommendations reasonably consistent with literature.	Measures tongue strength in 25 participants using IOPI in and confirms the presence of dysphagia in these participants using FEES.	The tongue strength test using the IOPI had a sensitivity of 91.67% and a specificity of 38.46% in detecting dysphagia as observed with FEES.
Carbó Perseguer et al. (2019) [11]: cross-sectional single center observation study	**3b:** Results consistent with literature; reasonably consistent recommendations; fairly definitive conclusions.	Describes the complications and mortality rates associated with the placement of percutaneous endoscopic gastrostomy tubes in individuals with ALS.	Percutaneous endoscopic gastrostomy tubes should be placed prior before patients are in the advanced stages of ALS or have excessively decreased forced vital capacity values.
Chapin et al. (2020) [12]: cross-sectional single center observation study	**3b:** Sufficient sample size for study design; reasonably consistent recommendations.	Investigates the discriminant ability of the ALSFRS-R bulbar subscale to detect dysphagia compared to videofluoroscopy in individuals with ALS.	For a cut-off score of ≤3 on the swallowing item of the ALSFRS-R bulbar subscale (which indicates dysphagia), sensitivity is 79% and specificity is 60%).
Donohue et al. (2022) [13]: cross-sectional single center observation study	**3b:** Recommendations reasonably consistent with literature; sufficient sample size for study design.	Examines the discriminant properties of the EAT-10 compared to videofluoroscopy in individuals with ALS.	A score of 3 on the EAT-10 indicated mild dysphagia while a score of 7 indicated moderate dysphagia as observed on videofluoroscopy.
Donohue et al. (2022) [13]: cross-sectional single center observation study	**3b:** Results reasonably consistent with literature; sufficient sample size for study design.	Examines the discriminant properties of the three-ounce water swallow test compared to videofluoroscopy in individuals with ALS.	The three-ounce water test had a sensitivity of 55% and a specificity of 72%. This screen should be used in combination with other measures.
Epps et al. (2020) [14•]: cross-sectional survey study	**3b:** Results reasonably consistent with literature; had fairly definitive conclusions; made recommendations consistent with findings.	Explores the current state of practice of speech-language pathologists in assessing and treating dysphagia in individuals with ALS.	The timing, methods, and recommendations of swallowing assessment, treatment, feeding tube placement, and other services vary among speech pathologists in the United States.
(Focht) Garand et al. (2021) [15]: cross-sectional multicenter observation study	**3b:** Results reasonably consistent with literature; had fairly definitive conclusions; made recommendations consistent with findings.	Validates the utility of the Yale Swallow Protocol in individuals with motor neuron disease including ALS, comparing Yale Swallow Protocol results to videofluoroscopy.	In individuals with motor neuron disease, the Yale Swallow Protocol has a sensitivity of 80% and a specificity of 33%, making it an adequate dysphagia screen.
McDonnell et al. (2017) [16]: retrospective data analysis	**3b:** Results reasonably consistent with literature; sufficient sample size for study design; reasonable recommendations.	Analyzed data retrospectively to examine the impact of gastric tube placement on survival and quality of life in individuals with ALS.	Gastric tube placement decreased survival time and had no effect on quality of life.
Onesti et al. (2017) [17]: retrospective observation cohort study	**3c:** Results reasonably consistent with literature; sufficient sample size.	Examines dysphagia progression in participants with bulbar and spinal onset ALS and its impact on nutritional management through medical chart review; also assesses riluzole impact on survival.	By the two-year follow-up, 82.8% of participants consisting of bulbar and spinal onset ALS had pharyngeal phase dysphagia according to FEES and PAS.
Parket al. (2022) [18]: cross-sectional single center observation study	**3b:** Results consistent with literature; recommendations reasonably consistent with literature; adequate sample size.	Compares the ALSFRS-R scores and videofluoroscopic findings of bulbar and spinal ALS onset types.	For both bulbar and spinal onset, aspiration and oral transit time were correlated with the ALSFRS-R bulbar subscale. Bulbar onset also had correlations with penetration.
Perry et al. (2018) [19]: cross-sectional single center observation study	**3b:** Results consistent with literature; recommendations reasonably consistent with literature.	Compared tongue and jaw kinematics of individuals with ALS with healthy participants during a 3-ounce water swallow task.	Abnormal findings in tongue and jaw speed, strength, range of motion, duration of movement, coordination, and efficiency can appear before overt signs of dysphagia.
Perry et al. (2021) [1]: large database study	**3b:** Results consistent with literature; recommendations consistent with findings; large sample size.	Estimates cumulative incidence of new onset dysphagia in large population consisting of both spinal and bulbar onset ALS.	Incidence of new dysphagia for bulbar onset ALS was 44% at 1 year and 64% at 2 years from study entry into clinical trial; it was 85% and 92%, respectively, for bulbar onset ALS.
Perry et al. (2021) [20]: cross-sectional single-center cohort observation study	**3b:** Results reasonably consistent with literature; fairly definitive conclusions; made reasonable recommendations.	Explores the relationship between biomechanical measures of the tongue such as speed and strength, and swallowing function using videofluoroscopy and patient self-report questionnaires.	Swallowing safety with unthickened liquids was affected by tongue speed and range of motion, and with pureed solids, by tongue strength.
Perry et al. (2022) [21]: large database study	**3b:** Results consistent with literature; made recommendations consistent with findings; large sample size.	Discusses the development and validation of a dysphagia risk for individuals with ALS based on clinically-accessible information such as ALSFRS subscale scores.	The healthcare team should consider swallowing evaluations for individuals with ALS who have dysphagia risk scores ranging from 15 to 50%
Plowman et al. (2017) [22]: cross-sectional survey study	**3b:** Results reasonably consistent with literature; made fairly definitive conclusions; made reasonably consistent recommendations.	Probes the current state of evaluating and managing speech and swallowing functions in clinical practice at ALS clinics.	Evaluating and managing ALS-related swallowing, including instrumental assessments and feeding tubes, is variable and inconsistent across ALS clinics in the United States.
Printzaetal. (2021) [23]: cross-sectional single-center observation study	**3b:** Results reasonably consistent with literature, fairly definitive conclusions; recommendations reasonably consistent with literature.	Measures tongue strength using IOPI, the degree of swallowing impairment using the EAT-10 and ALSFRS-R, and the amount of pharyngeal residue using the Yale Pharyngeal Residue Severity Rating Scale, and confirms the presence of aspiration using FEES.	An EAT-10 score of 8 (sensitivity: 100%, specificity: 42.90%), a maximum isometric tongue pressure of 22 KPa (sensitivity: 80%, specificity: 89.50%) and a Yale Pharyngeal Residue Severity Rating Scale score of 1 (sensitivity: 90%; 80%) predict aspiration on FEES.
Seeber et al. (2019) [24]: qualitative study	**3b:** Results consistent with literature; made fairly definitive conclusions.	Discusses the process and patient perspectives of advance care planning for individuals with ALS and progressive muscular atrophy.	Advance care planning, including nutritional management, allows healthcare providers to be aware of the changing wishes of the patient as the disease progresses.
Smith et al. (2018) [25]: non-experimental study	**3b:** Results reasonably consistent with literature; sufficient sample size for the study design; fairly definitive conclusions; recommendations reasonably consistent with literature.	Validates the psychometric properties of the Center for Neurologic Study Bulbar Function Scale in individuals with ALS who are or are not taking Nuedexta.	The measure has a test-retest reliability of 0.86; it also has a sensitivity of 83% and specificity of 72% when detecting patient-reported swallowing abnormalities.
Tabor-Gray et al. (2020) [26]: cross-sectional multicenter observational study	**3b:** Results reasonably consistent with literature; recommendations reasonably consistent with literature.	Examines the differences between reflexive and voluntary coughing in individuals with ALS and healthy participants.	Reflexive coughing in response to capsaicin stimulus was approximately less than half as strong compared to voluntary coughing in individuals with ALS.
Tyeetal. (2021) [27]: retrospective cross-sectional single center observational study	**3b:** Results reasonably consistent with literature; sufficient sample size; recommendations reasonably consistent with literature.	Discusses fiberoptic endoscopic evaluation results in individuals with varying neurodegenerative diseases, including ALS.	Individuals who score low on self-reported swallowing measures, like the Eating Assessment Tool-10, can have unremarkable instrumental results.
Zarotti et al. (2019) [28]: qualitative study	**3b:** Results consistent with literature; sufficient sample size.	Explores barriers and facilitators to nutritional management of individuals with ALS from the viewpoints of various healthcare providers.	Psychological adjustment, engagement after diagnosis, need for control, knowledge of nutrition, and psychosocial eating factors impact participation in nutrition plans.
**Level 4**			
No studies have this level of evidence.			
**Level 5**			
Rogus-Pulia & Plowman (2020) [29••]: expert opinion	**5a:** Though leaders in the field; expertise appears to be credible; draws definitive conclusions; provides scientific rationale.	Advocates for proactive care in the assessment and treatment of dysphagia in individuals with motor neuron diseases like ALS.	Proactive assessment and treatment of dysphagia in motor neuron diseases employs patient-centered interdisciplinary team care with SLPs as early as ALS diagnosis.
Yunusova et al. (2019) [30]: literature review	**5a:** Expertise is clearly evident; draws definitive conclusions; provides scientific rationale; thought leaders in the field.	Overviews common screens and evaluations currently used to assess speech and swallowing over varying time periods in individuals with ALS.	Although there are a number of new assessment tools, only a few meet basic measurement requirements of inter- and intra-rater reliability, sensitivity, specificity, and responsiveness.
Britton et al. (2018) [31]: literature review	**5b:** Expertise appears to be credible; draws definitive conclusions; provides logical argument for opinions.	Provides a general overview of assessment, treatment, and management strategies of neuromuscular diseases, not just ALS.	Individuals with progressive diseases such as ALS should receive periodic assessments and updated recommendations for swallowing.
Everett et al. (2020) [32]: literature review	**5b:** Expertise appears to be creditable; draws fairly definitive conclusions; provides logical argument for opinions.	Provides a general overview of the care of individuals with ALS geared for clinicians working in palliative care.	Clinicians in palliative care should be aware of symptoms, disease course, treatments, and advance care planning considerations for individuals with ALS.
Marques et al. (2018) [33]: literature review	**5b:** Draws fairly definitive conclusions; provides logical argument for opinions.	Provides a brief overview of the benefits of percutaneous gastrostomy tube feeding as well as the optimal timing of placement in individuals with ALS.	Early placement of gastrostomy tube may be beneficial for individuals with ALS by delaying debilitation and allowing the individual to better respond to interventions.
Soga et al. (2022) [34]: case series	**5b:** Draws fairly definitive conclusions; provides logical argument for opinions.	Discusses the benefits and risks of central-part laryngectomy on speech and swallowing in individuals with ALS.	Central-part laryngectomy may be an effective option for reducing aspiration risk in patients in the late stages of ALS, allowing oral intake for up to 2–4 years after surgery.
Waito et al. (2020) [35]: scoping review	**5b:** Clear aims and objectives; formal quality evaluation methods used; reasonably consistent recommendations; refers to scientific evidence.	Discusses trends of dysphagia research in motor neuron diseases including ALS.	The most commonly researched topics were aspiration/penetration and enteral nutrition, with the caveat that only articles published in English peer-reviewed journals were included.

*ALSFRS-R* Amyotrophic Lateral Sclerosis Functional Rating Scale Revised; *EAT-10* Eating Assessment Tool-10; *EMST Expiratory* Muscle Strength Training; FEES fiberoptic endoscopic evaluation of swallowing; *IOPI* Iowa Oral Performance Instrument; *PAS* Penetration Aspiration Scale; *PEG* percutaneous endoscopic gastrostomy; *SLP* speech-language pathologists

**Table 2:** Sensitivity and specificity of evaluations in detecting penetration and aspiration confirmed with videofluoroscopy

Sensitivity and Specificity of Select Assessment Tools				
Tool	Sensitivity	Specificity	AUC	Interpretation
3-oz Water Swallow (penetration)	Not assessed [15]	Not assessed [15]	Not assessed [15]	These psychometric properties have not yet been validated in individuals with ALS.
	Not assessed [44]	Not assessed [44]	Not assessed [44]
3-oz Water Swallow (aspiration)	80% [15]	33.3% [15]	Not assessed [15]	Based on (Focht) Garand et al. (*n* = 31), you will miss 20% true positives and 66.7% of true negatives. Based on Donohue et al. (*n* = 212), you will miss 44.8% of true positives and 28.3% of true negatives. The AUC reported by Donohue et al. is poor.
	55.2% [44]	71.7% [44]	0.64 [44]	
EAT-10 (penetration/aspiration)	88% [43]	57% [43]	0.77 [43]	You will miss 12% of true positives and 43% of true negatives. The AUC is fair.
EAT-10 (aspiration)	86% [43]	72% [43]	0.88 [43]	You will miss 14% of true positives and 28% of true negatives. The AUC is good.
ALSFRS-R (penetration/aspiration)	79% [12]	60% [12]	0.72 [12]	You will miss 21% of true positives and 40% of true negatives. The AUC is fair.
CNS-BFS (aspiration/penetration)	Not assessed [25]	Not assessed [25]	Not assessed [25]	These psychometric properties have not yet been validated in individuals with ALS.

*EAT-10* Eating Assessment Tool-10; *ALSFRS-R* ALS Functional Rating Scale Revised; *CNS-BFS* Center for Neurological Study Bulbar Function Scale

*AUC values Excellent*: 90−100; *Good* 80−90; *Fair* 70−80; *Poor* 60−70; *Fail* 50−60 [45]
